# Biomarkers for Precision Prognosis in Prostate Cancer: Imaging, Molecular, and Integrated Approaches

**DOI:** 10.3390/cancers18111751

**Published:** 2026-05-27

**Authors:** Zahra Khazaei, Frédéric Pouliot, Louis Archambault

**Affiliations:** 1 Département de Physique, de Génie Physique et D’optique, Faculté des Sciences et de Génie, Université Laval, Québec, QC G1V 0A6, Canada; zahra.khazaei.1@ulaval.ca; 2 Axe Oncologie, Centre de Recherche du CHU de Québec–Université Laval, Québec, QC G1V 4G2, Canada; frederic.pouliot@crchudequebec.ulaval.ca

**Keywords:** prostate cancer, prognosis, imaging biomarkers, non-imaging biomarkers, radiomics, artificial intelligence, precision oncology, risk stratification

## Abstract

Prostate cancer (PCa) is predominantly an acinar adenocarcinoma arising from the prostatic glandular epithelium, with tumor grade assessed using the International Society of Urological Pathology (ISUP) Grade Group classification, reflecting the degree of glandular differentiation and underlying molecular heterogeneity. PCa exhibits wide clinical behavior heterogeneity, ranging from indolent disease to aggressive forms with poor outcomes. Accurate prognostic assessment is, therefore, essential for guiding treatment selection and monitoring disease progression. This review examines recent advances in imaging and non-imaging biomarkers that contribute to improved risk stratification, treatment planning, and disease monitoring. Particular attention is given to multiparametric magnetic resonance imaging (mpMRI), whole-body magnetic resonance imaging (WB-MRI), positron emission tomography/computed tomography (PET/CT), positron emission tomography/magnetic resonance imaging (PET/MRI), computed tomography (CT), and transrectal ultrasound (TRUS), evaluated for their capacity not only to detect disease but also to predict recurrence, progression, and survival outcomes. In parallel, the prognostic role of non-imaging biomarkers is discussed, including the prostate-specific antigen (PSA), histopathological grading, biochemical and inflammatory biomarkers, as well as genomic classifiers and circulating tumor DNA (ctDNA). Emerging approaches such as radiomics, liquid-biopsy-derived molecular profiles, and artificial intelligence (AI)-based multimodal integration are highlighted for their potential to enhance individualized decision making. This review underscores the importance of combining imaging and molecular information to refine prognostic models and accelerate the translation of precision medicine in PCa.

## Simple Summary

Prostate cancer can range from harmless tumors to aggressive disease that can be highly heterogeneous. Doctors need better ways to tell which patients require immediate treatment and which can be safely monitored. This review explains how current medical imaging tools such as MRI, PET, CT, and ultrasound, together with blood tests, tissue studies, and genetic information, are being used to improve prostate cancer prognosis and its evolution. By combining these approaches, researchers are moving toward more precise and personalized care. This means that patients could receive treatments tailored to their individual disease, while avoiding unnecessary procedures. The review also highlights how new technologies, such as artificial intelligence and advanced computer analysis, may further improve decision making. Together, these advances could help doctors provide safer and more effective treatments for men with prostate cancer.

## 1. Introduction

Prostate cancer (PCa) is the second most commonly diagnosed cancer in men worldwide according to 2022 global estimates, and the most frequently diagnosed malignancy among men in nearly two-thirds of the world’s countries (118 of 185) [1], with a broad range of clinical outcomes, ranging from indolent tumors that may never require intervention to highly aggressive forms that demand immediate and multifaceted treatment. Accurate prognosis is crucial for guiding clinical decisions, particularly in selecting appropriate treatment strategies and monitoring disease progression. Advances in imaging technologies have played a key role in enhancing our ability to predict patient outcomes, moving beyond traditional diagnostic methods to incorporate detailed anatomical and functional insights [2,3].

Magnetic resonance imaging (MRI) plays an important role in prostate cancer management due to its ability to provide high-resolution images and functional data that support disease detection, staging, and risk stratification [4,5,6]. The development of multiparametric MRI (mpMRI) in recent years has further expanded its clinical utility, enabling more accurate biopsy targeting and improved assessment of tumor aggressiveness [6,7,8].

Meanwhile, positron emission tomography (PET), particularly when combined with computed tomography (CT), has emerged as another critical tool, especially for detecting metastatic disease and recurrent cancer [9]. PET imaging with prostate-specific tracers, such as prostate-specific membrane antigen (PSMA), has demonstrated superior sensitivity and specificity compared with many traditional methods in specific clinical contexts [10,11,12].

Transrectal ultrasound (TRUS) remains widely used, particularly for guiding biopsies; however, its prognostic value is relatively limited compared to MRI [13]. Nonetheless, integrating TRUS with advanced imaging modalities such as PET and MRI can provide complementary diagnostic insights [14,15].

**Figure 1:**
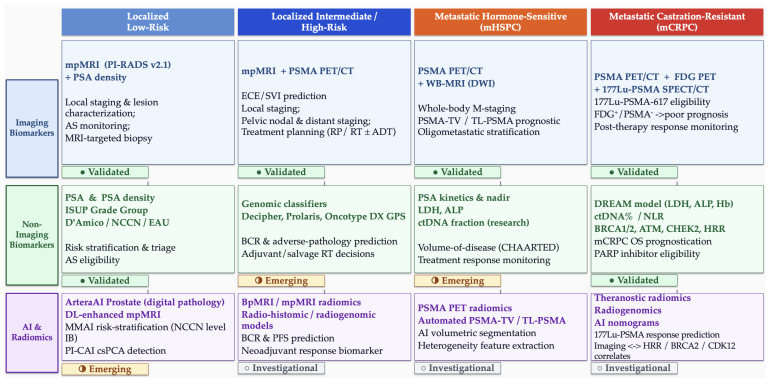
Framework for integrating imaging, non-imaging, and AI-derived biomarkers across prostate cancer disease stages. Biomarkers are organized by domain (rows) and mapped to four disease states (columns): localized low-risk, localized intermediate/high-risk, metastatic hormone-sensitive (mHSPC), and metastatic castration-resistant (mCRPC). Evidence levels: • guideline-endorsed (EAU 2024/NCCN v4.2024) or prospective RCT-validated; ◐ commercially available with multicenter evidence; ∘ investigational. Imaging anchors: mpMRI (PI-RADS v2.1) [18,19,20]; PSMA PET/CT for staging [12]; FDG/PSMA discordance and ^177^Lu-PSMA-617 in mCRPC [21,22]. Non-imaging anchors: Decipher genomic classifier (NCCN level IB) [23]; CHAARTED volume criteria for mHSPC [24]; PARP-inhibitor eligibility via HRR alterations [25]; DREAM model for mCRPC OS [26].

Beyond imaging, the integration of genomic, molecular, and clinical data with imaging findings represents a paradigm shift in personalized medicine for PCa [3,16,17]. This multimodal approach could offer a more thorough understanding of the disease, enabling clinicians to tailor treatments more effectively and better predict outcomes. In this review, we discuss the roles of MRI, CT, PET, and TRUS in PCa prognosis, emphasizing their integration with non-imaging data, including clinical records, genomic markers, and molecular profiles. By synthesizing insights from various modalities, this review aims to critically evaluate recent advancements and their implications for the future of PCa management. Figure 1 illustrates how imaging, non-imaging, and AI-based biomarkers map onto different stages of PCa, from localized disease through metastatic progression.

## 2. Prognostic Markers in Prostate Cancer

Imaging modalities such as mpMRI, PET/CT, and bone scintigraphy (BS) form the backbone of PCa staging and surveillance, yet each carries limitations: BS, although widely accessible, shows substantially lower sensitivity and specificity than PSMA PET/CT [12], while mpMRI and PET/CT achieve higher accuracy but are constrained by cost, uneven access, and persistent false negatives for small or multifocal lesions [2,27].

Given these challenges, there is growing recognition that imaging alone may not provide a comprehensive prognostic picture. Integrating non-imaging biomarkers, including clinical, biochemical, metabolic, histopathological, and genomic markers, offers a complementary perspective that can improve risk stratification, personalize treatment, and ultimately optimize outcomes in PCa [3,17,28].

Risk stratification is essential to PCa management, ensuring that treatment decisions are tailored to each patient’s risk profile. For example, patients with low-risk PCa are increasingly managed with active surveillance, a strategy supported by evidence that such tumors often progress slowly or remain indolent. Consequently, active surveillance is sometimes favored over aggressive interventions like radiotherapy (RT) or prostatectomy, especially when evidence suggests that low-risk tumors may not impact overall mortality [29]. According to a nationwide study, approximately 60% of US men diagnosed with low-risk PCa are now managed with active surveillance. The rate of active surveillance for low-risk PCa has more than doubled between 2014 and 2021, increasing from 26.5% to 59.6% [30]. Conversely, early identification of high-risk cases is important for closer monitoring and precise therapeutic interventions [31]. Therefore, developing and refining tools to categorize patients into distinct risk groups remains a priority for improving patient outcomes.

One of the most established frameworks for classifying PCa risk is the D’Amico Risk Classification system [32], which categorizes PCa patients into low-, intermediate-, and high-risk groups based on clinical stage, serum prostate-specific antigen (PSA) levels, and Gleason score from biopsies—corresponding to ISUP grade group (GG) in contemporary practice. Although widely used, several other risk stratification tools have been developed to address its limitations and improve its accuracy, including grouping systems that incorporate more detailed clinicopathological data or additional parameters, as well as risk scores and nomograms. These include frameworks such as the National Institute for Health and Care Excellence (NICE), European Association of Urology (EAU), American Urological Association (AUA), and National Comprehensive Cancer Network (NCCN), that integrate more detailed clinicopathological data (e.g., ISUP GGs, biopsy core involvement) to improve prognostic accuracy [5,23,33,34]. For example, a nationwide cohort study of 139,515 Swedish men found that risk models like the Memorial Sloan Kettering Cancer Center (MSKCC) nomogram [35], the Cancer of the Prostate Risk Assessment (CAPRA) score [36], and the Cambridge Prognostic Groups (CPG) [37] outperformed the D’Amico system in predicting prostate cancer-specific mortality. Specifically, at 10 years of follow-up, the MSKCC nomogram (C-index 0.81; 95% confidence interval (CI) 0.80–0.81), the CAPRA score (C-index 0.80; 95% CI 0.79–0.81), and the CPG system (C-index 0.78; 95% CI 0.78–0.79) outperformed the D’Amico system (C-index 0.73; 95% CI 0.72–0.73) in predicting PCa-specific mortality at 10 years [38]. A comparative summary of these risk stratification frameworks is provided in Table 1.

Despite advances in clinical risk models, pretreatment PCa risk assessment remains challenging, leading to the exploration of new techniques that utilize genomic markers to enhance accuracy. At the forefront of these developments are tissue-based technologies, which aim to overcome the inherent difficulties of existing risk assessment techniques to improve prognostic models and patient outcomes [39,40].

In addition, traditional diagnostic methods such as tissue biopsy, the gold standard for diagnosis, face limitations including invasiveness, tumor heterogeneity, and sampling errors [15,41]. This has led to increased interest in liquid biopsy as a non-invasive alternative for evaluating circulating biomarkers, with the potential to further enhance prognostic accuracy and guide personalized treatment strategies [42,43].

### 2.1. Clinical and Biochemical Markers

Clinical and biochemical markers are key in the risk stratification and prognosis of PCa. These markers are accessible and cost-effective diagnostic and monitoring tools compared to imaging and genomic assays.

#### 2.1.1. PSA and Its Prognostic Role

Among clinical biomarkers, PSA remains the most widely utilized, guiding screening, tumor burden estimation, and treatment response assessment [39].

A large-scale analysis by the Norwegian Prostate Cancer Consortium (NPCC), based on over 8 million PSA measurements in men aged 40–69 years, identified baseline serum PSA levels as strong predictors of long-term PCa mortality. Even moderately elevated PSA levels (2.0–3.9 ng/mL), below the traditional biopsy threshold, were associated with significantly increased mortality over a 15–20 year period [44].

The prognostic value of PSA has also been demonstrated in the therapeutic context. In patients receiving ^177^Lu-PSMA-617 for metastatic castration-resistant prostate cancer (mCRPC), significant PSA reductions (≥50% or ≥90%) were associated with improved survival outcomes [45].

#### 2.1.2. PSA Dynamics and Biochemical Recurrence

Beyond baseline PSA levels, longitudinal PSA behavior provides additional prognostic information, particularly in the post-treatment setting. Biochemical recurrence (BCR), defined by a rise in PSA following primary therapy, remains a key endpoint for monitoring disease progression [46]. However, it is important to note that the definition of BCR varies substantially according to the treatment modality. Following radical prostatectomy (RP), BCR is typically defined as a confirmed prostate-specific antigen (PSA) level of ≥0.2 ng/mL [34]. In contrast, after RT, BCR is defined according to the Phoenix criterion as a PSA increase of ≥2.0 ng/mL above the post-treatment nadir [47]. These distinct definitions limit direct comparisons of recurrence rates across treatment strategies.

PSA kinetics, including doubling time and nadir PSA levels, are critical in predicting disease progression and tailoring treatment strategies [48,49,50]. The utility of PSA dynamics is especially evident in the post-treatment setting. PSA nadir measured six months after RT is a robust prognostic marker. A pooled analysis of 16 randomized trials involving 10,415 patients revealed that PSA levels ≥ 0.1 ng/mL at nadir were associated with significantly worse outcomes across all treatment groups (RT alone, RT + short-term androgen deprivation therapy (ADT), and RT + long-term ADT), including metastasis-free survival (MFS), prostate cancer-specific mortality (PCSM), and overall survival (OS) [51]. For instance, five-year MFS rates for RT alone were 91% versus 79% for low versus high PSA nadir, respectively.

Despite its established use, controversy remains regarding optimal PSA thresholds and timing for intervention in BCR. There is no universal consensus among guideline associations on the PSA level that should prompt salvage therapy after RP [52]. Moreover, differences in treatment strategies, including the use of ADT in intermediate- and high-risk disease, complicate the interpretation of PSA-based recurrence endpoints across modalities. This variability highlights the need for standardized reporting frameworks and raises debate about the clinical significance of low-detectable PSA values [49,53,54].

When PSA levels signal recurrence, imaging is required to localize disease before salvage therapy can be planned. PSMA PET/CT has transformed this workup, detecting sites of recurrence at PSA thresholds well below those at which conventional CT and bone scintigraphy typically become informative [55,56].

#### 2.1.3. Beyond PSA: Inflammatory and Coagulation Markers

In addition to PSA, other systemic markers enhance PCa prognosis. Serum-based markers such as fibrinogen (Fib), alkaline phosphatase (ALP), lactate dehydrogenase (LDH), and protein signatures like osteopontin and L-selectin have demonstrated utility in grading severity and predicting tumor aggressiveness [57,58].

Elevated fibrinogen (Fib) levels independently predict high-risk disease (odds ratio, OR = 15.2, p=0.014) and are strongly associated with advanced clinical stage, ISUP GG 4–5, and metastatic disease [59].

Inflammatory markers like neutrophil-to-lymphocyte ratio (NLR) and C-reactive protein (CRP) are also associated with adverse outcomes. Elevated NLR has been linked to disease progression and poor survival, particularly in mCRPC [60], while high pretreatment CRP levels are correlated with extracapsular extension, lymph node involvement (LNI), and higher recurrence risk [61,62].

#### 2.1.4. Multimarker and Multimodal Integration for Risk Stratification

Integrating multiple biomarkers can improve the robustness of prognostic models. In the Prostate Cancer DREAM (Dialogue for Reverse Engineering Assessments and Methods) Challenge, LDH, hemoglobin, and alkaline phosphatase were consistently identified as key predictors of overall survival in mCRPC. The top-performing ensemble model (ePCR) stratified patients into distinct risk groups with strong prognostic separation (hazard ratio (HR) = 3.32, p<0.0001) [26].

Similarly, the CRISP3/SPINK1 prognostic grade (CSPG), detected in expressed prostatic secretion urine, predicted BCR (HR = 3.14, *p* < 0.001) and cancer-specific survival, with an area under the receiver operating characteristic curve (AUC) of 0.87 at 5 years [63].

Beyond serum and urinary markers, recent studies have highlighted the value of integrating imaging features with biochemical indicators. In a prospective cohort of 108 participants, restriction spectrum imaging–MRI (RSI-MRI) outperformed the Prostate Health Index (PHI) for predicting upgrading on active surveillance (AS) biopsies (AUC 0.84 vs. 0.68). While PHI and PSA density had limited standalone utility, their combination with imaging features improved overall discrimination, supporting complementary use to inform biopsy strategies and reduce overtreatment in localized disease [64].

A multicenter diagnostic model integrating imaging and biochemical markers for clinically significant prostate cancer (csPCa), combining PI-RADS v2.1 from mpMRI with prostate-specific antigen density (PSAD), demonstrated excellent discrimination across development and external validation cohorts (AUCs 0.941, 0.862, and 0.896). Risk stratification using predefined probability thresholds and decision curve analysis showed that up to 45.6% of prostate biopsies could be avoided at a low-risk threshold, with less than 1% of csPCa cases missed, while maintaining high sensitivity across cohorts [65].

According to the EAU Prostate Cancer Guidelines, several validated blood-based and multivariable risk stratification models have been proposed to improve the detection of clinically significant prostate cancer and reduce unnecessary biopsies. These include the PHI, the 4Kscore test, IsoPSA, the Stockholm3 model, and Proclarix, all of which have demonstrated improved discrimination over PSA alone and complementary value when combined with mpMRI [66]. Similarly, a multicenter study in Saudi Arabia developed and internally validated a nomogram for csPCa in men with elevated PSA and suspicious MRI findings. Incorporating PI-RADS, prostate volume, and total PSA, the tool yielded an AUC of 0.83 with good calibration; decision curve analysis suggested about a 5% biopsy reduction at a 30% probability threshold without missing csPCa [67].

Taken together, these results reaffirm that while PSA remains essential, integrated models combining traditional clinical markers (PSA, Gleason grade) with advanced imaging features and validated prognostic tools can deliver more precise risk estimation and reduce unnecessary interventions. Standardizing the clinical integration of these markers, while addressing accessibility and cost barriers, will be critical to optimizing personalized care in prostate cancer.

### 2.2. Clinical Nomograms and Comorbidity Burden

In addition to biomarkers and imaging, patient-level clinical context can influence prognosis and management.

An analysis of conservatively managed PCa patients using eight international datasets encompassing over 100 million individuals highlighted the influence of comorbidities and age on clinical outcomes. In a cohort of 123,146 patients, conditions such as hypertension (35–73%) and type 2 diabetes (11–28%) significantly impacted the risks of symptomatic progression, hospitalization, and emergency department visits. Kaplan–Meier analysis revealed that the need for curative or palliative treatment declined over time, underscoring the value of real-world data in guiding PCa management and informing clinical decision making [68].

Clinical nomograms can also provide individualized risk estimates that complement biomarker-based approaches. Early models such as the Briganti nomogram demonstrated the utility of clinical variables for predicting LNI [69]; however, subsequent updates have improved performance by incorporating modern imaging. The Gandaglia nomogram integrates mpMRI findings—specifically the maximum diameter of the index lesion—alongside PSA, clinical stage, targeted biopsy grade group, and the presence of clinically significant cancer on concomitant systematic biopsy, achieving an AUC of 0.86 for predicting LNI and outperforming earlier models in MRI-targeted biopsy populations [70]. EAU guidelines recommend the use of validated nomograms incorporating MRI findings to guide the indication for extended pelvic lymph node dissection [66], exemplifying the added value of multimodal integration in risk stratification.

### 2.3. Metabolic Biomarkers

Metabolic biomarkers offer a promising avenue for enhancing the diagnosis, prognosis, and management of PCa. The healthy prostate is characterized by a distinctive metabolic profile, marked by high concentrations of citrate and zinc, which are notably disrupted during malignant transformation [71]. This metabolic shift, in conjunction with the influence of factors such as metabolic syndrome on PCa risk, underscores the potential of metabolite profiling to elucidate the biological processes of PCa [71].

Recent advancements in metabolomics have led to the identification of several promising biomarkers. Alterations in polyamines, tricarboxylic acid (TCA) cycle intermediates, amino acids, and fatty acids have been consistently reported in PCa [72]. For instance, metabolites such as sarcosine, choline, and phosphocholines have demonstrated potential in distinguishing PCa cases from healthy controls [73]. Additionally, a panel of 18 inflammatory and cytokine serum biomarkers, including vascular endothelial growth factor (VEGF) and interleukin-6 (IL-6), has shown enhanced detection of aggressive PCa [57]. The integration of metabolomic data with genetic markers, as exemplified by the Michigan Prostate Score 2 (MiPS2), has further improved diagnostic accuracy, while exosome-based biomarkers (e.g., prostate cancer antigen 3 (PCA3), SAM pointed domain-containing ETS transcription factor (SPDEF), and transmembrane protease, serine 2–ETS-related gene fusion (TMPRSS2-ERG)) offer a non-invasive approach to profiling tumor RNA (ribonucleic acid) expression [57].

Metabolite biomarkers also exhibit prognostic potential. Metabolomic profiles derived from histologically benign tissues can predict PCa aggressiveness and recurrence, providing prognostic information that may exceed that of current gold-standard methods [71,74]. Elevated glutamate levels, for example, have been observed with high diagnostic performance (AUC of 90%) in distinguishing PCa from benign prostatic hyperplasia [75].

Furthermore, distinct changes in choline, lysophosphatidylcholines, and amino acids (e.g., aspartate, ornithine, serine, proline) have been documented between PCa and control groups [72,76], while a panel of volatile organic compounds, including hexanal and 2,5-dimethylbenzaldehyde, has achieved 89% sensitivity and 83% specificity for PCa identification [77]. These findings, along with observed alterations in polyamine and fatty acid metabolism, underscore the multifaceted role of metabolic biomarkers in improving risk stratification and guiding clinical management [71,75].

Peng et al. developed a diagnostic approach combining prostate fluid metabolic fingerprints with PSA levels using a ferric nanoparticle–assisted laser desorption/ionization mass spectrometry platform. In their cohort, the model achieved excellent diagnostic performance for identifying high-grade disease (ISUP GG ≥ 4), with a reported AUC of 0.96, sensitivity of 100%, and specificity of 88% [78]. In parallel, urinary oncometabolites, such as a panel including sarcosine, kynurenine, and ethanolamine, have demonstrated significant correlations with tumor aggressiveness and hold promise for refining prognostic assessments [79].

Some of these metabolic shifts have direct imaging counterparts. The elevated choline and phosphocholine levels identified in tissue and liquid metabolomics form the biological rationale for ^11^C- and ^18^F-choline PET/CT imaging, where increased tracer uptake reflects the same membrane phospholipid turnover active in malignant prostate tissue [80,81].

### 2.4. Histopathological Grading

Histopathological grading remains one of the most reliable predictors of PCa outcomes, particularly in patients with high-risk disease. Importantly, high-risk prostate cancer represents a heterogeneous group, with substantial variation in prognosis depending on the defining features.

In a large retrospective study of 1374 men treated with modern RT, Yamazaki et al. differentiated between high-risk low-grade (HRLG, Gleason ≤6, i.e., ISUP GG 1) and high-risk high-grade (HRHG, Gleason ≥7, i.e., ISUP GG ≥2) groups. The HRLG cohort showed superior outcomes, including higher 5-year biochemical disease-free survival (97.8% vs. 91.8%, p=0.017), no clinical failures (0% vs. 5.4%, p=0.012), and better 5-year distant metastasis-free survival (100% vs. 96.0%, p=0.035). Notably, no PCa-related deaths occurred in the HRLG group, whereas all such events (*n* = 16) were confined to HRHG patients. On multivariate analysis, low-grade histology (ISUP GG 1) remained an independent prognostic factor for biochemical disease-free survival (HR 0.20; 95% CI 0.05–0.83; p=0.027) [82].

These findings are consistent with earlier evidence demonstrating marked heterogeneity within high-risk prostate cancer definitions. In a multi-institutional cohort of 6477 men undergoing radical prostatectomy, Mossanen et al. showed that high-grade disease (biopsy Gleason score 8–10, corresponding to ISUP GG 4–5) and advanced clinical stage (T3) were the strongest predictors of prostate cancer–specific mortality, with hazard ratios approaching 18–20, whereas PSA-based high-risk definitions were associated with substantially lower mortality risk [83].

Another large study involving 2003 patients with localized PCa from the PROCURE biobank demonstrated that the ISUP-recommended 5-tier Gleason grading system reliably predicts recurrence-free survival, metastasis, castration-resistant prostate cancer (CRPC), and cancer-specific mortality over a median follow-up of 8.7 years. Notably, the authors proposed a refined 6-tier GG system, by upgrading patients with minor or tertiary Gleason pattern 5 in Gleason 3 + 4 and 4 + 3 tumors and further stratifying GG5 by the primary Gleason pattern, which outperformed the traditional 5-tier system, as evidenced by higher Harrell’s concordance indices (0.853 for CRPC-FS and cancer-specific mortality) [84].

Cimadamore et al. addressed several questions regarding the Gleason grading system and its prognostic implications. Their work chronicled the evolution of grading, from the original architectural classification to the updated ISUP systems of 2014 and 2019, which introduced five distinct GGs (GG1 through GG5). The study underscored the prognostic significance of cribriform morphology, noting that patients with cribriform features, as well as those with intraductal carcinoma of the prostate (IDC-P), experience poorer outcomes, including increased rates of metastasis, BCR, and adverse surgical margins. The authors also highlighted the benefits of targeted biopsies and molecular testing in minimizing grading discrepancies and enhancing prognostic precision [85].

Cribriform morphology has also been identified as an independent prognostic marker for BCR in patients with high-grade disease. In patients with ISUP GG 4 (corresponding to Gleason score 4 + 4), Shimodaira et al. demonstrated that a higher proportion of cribriform architecture was significantly associated with increased risk of BCR. Multivariate analysis confirmed that both preoperative PSA levels and the percentage of cribriform pattern were independent predictors of recurrence. Patients with cribriform involvement exceeding 50% experienced substantially lower 3-year biochemical recurrence-free survival (BCR-FS) compared with those without cribriform patterns [86].

The prognostic significance of IDC-P has also been highlighted by Kato et al. Their retrospective analysis of 145 patients undergoing RP with neoadjuvant ADT revealed that persistence of IDC-P is associated with significantly worse disease-free survival (DFS), CSS, and OS compared to cases where IDC-P was absent or had disappeared post-treatment. Multivariate Cox regression further identified IDC-P persistence as an independent predictor of poor prognosis [87].

The heterogeneity observed within very high-grade prostate cancer underscores the need for detailed histopathological grading. A population-based study by Egevad et al. including 20,419 men in Sweden demonstrated marked differences in 10-year prostate cancer-specific mortality among patients classified as ISUP GG 5 (corresponding to Gleason scores 9–10). Mortality rates were 66% for Gleason 5 + 5, 56% for Gleason 5 + 4, and 45% for Gleason 4 + 5. Multivariable analyses confirmed that both Gleason 5 + 4 and 5 + 5 patterns were independent predictors of poor outcomes, highlighting the importance of preserving detailed Gleason pattern information within ISUP GGs to avoid oversimplification that could obscure clinically relevant prognostic differences [88].

Finally, Zhanghuang et al. utilized data from the Surveillance, Epidemiology, and End Results (SEER) database to develop nomograms that predict cancer-specific and overall survival in elderly patients based on factors such as age, PSA levels, Gleason score, and treatment modality. Their models, which demonstrated high predictive accuracy (c-indices of 0.802 for cancer-specific survival (CSS) and 0.712 for OS), provide valuable tools for personalized treatment planning in this population [89].

Histopathological grading is only as reliable as the tissue to which it is applied. Systematic biopsy, which samples the gland without anatomical guidance, is prone to grade underestimation due to tumor heterogeneity. mpMRI-targeted biopsy directs sampling to index lesions identified by PI-RADS scoring, yielding better detection of high-grade disease and fewer low-grade overdiagnoses compared with systematic cores alone [18,19]. The grade assigned from MRI-targeted specimens, therefore, carries stronger prognostic weight and more accurately reflects the tumor phenotype relevant to treatment planning. Beyond conventional grading, multimodal AI-based digital-pathology tools have begun to augment histopathological assessment: the 2024 NCCN Prostate Cancer Guidelines (Version 3.2024) tabulate the ArteraAI Prostate model at Simon level of evidence IB for both predictive and prognostic use, distinguishing it from exploratory deep-learning pathology tools that have not yet reached comparable validation [23].

### 2.5. Genomic Markers

In recent years, genomic markers have become increasingly important for refining the prognosis of PCa, as they can reveal the molecular basis of tumor behavior. Unlike traditional imaging or clinical biomarkers, genomic data provide insight into complex genetic and molecular pathways behind progression. For example, Li et al. identified autophagy-related long non-coding RNAs (au-lncRNAs) as significant prognostic biomarkers, and their risk model effectively stratified patients into high- and low-risk groups that correlated with disease-free survival [90]. Similarly, Shore et al. reviewed evidence that the Decipher genomic classifier enhances risk stratification for patients with BCR by integrating molecular profiles with clinical markers such as PSA [53]. Reflecting this maturing evidence base, the 2024 NCCN Prostate Cancer Guidelines (Version 3.2024) now formally tabulate the 22-gene Decipher genomic classifier at Simon level of evidence IB, the highest tier achievable with prospective–retrospective validation, distinguishing it from other genomic assays such as the 31-gene cell-cycle progression panel (Prolaris) and the 17-gene Genomic Prostate Score, which currently sit at lower evidence tiers [23].

Further, Wang et al. introduced a novel prognostic model that combined Gleason grading with cuprotosis-related gene expression. Using data from The Cancer Genome Atlas–Prostate Adenocarcinoma (TCGA-PRAD) and GSE70769, they identified five critical genes, STX3, CABLES2, E2F5, RALA, and POLE3, associated with disease-free survival. Their model, which stratified patients into distinct risk groups with AUCs of 0.81, 0.79, and 0.72 for 1-, 3-, and 5-year survival, respectively, also highlighted STX3 as a promising therapeutic target [91].

Moreover, the integration of genetic and clinical data through machine learning (ML) has further improved prognostic predictions. In the KYUCOG-1401-A study, Shiota et al. developed a predictive model for castration resistance by combining clinical parameters and single-nucleotide polymorphism (SNP) data, achieving concordance indices (C-indices) above 0.7 for progression-free, cancer-specific, and overall survival [92].

Lehto et al. further characterized MRI-visible prostate lesions. Their integration of histomic and transcriptomic data from 45 RP patients revealed that MRI-visible lesions exhibit transcriptomic signatures of increased proliferation and inflammation, correlating with poorer metastasis-free and cancer-specific survival [93]. In the liquid biopsy arena, Fonseca et al. validated circulating tumor DNA fraction (ctDNA%) as a robust prognostic tool in mCRPC, showing that ctDNA% is strongly associated with overall survival and treatment response, independent of conventional clinical markers [94].

Advances in programmed cell death research have also yielded the Programmed Cell Death Index (PCDI), an 11-gene signature developed by Wang et al. that robustly predicts BCR across multiple datasets. High PCDI values correlate with advanced tumor stage, reduced sensitivity to agents such as Docetaxel, and poorer immunotherapeutic response, whereas lower values are indicative of a better immune response [95]. Additionally, Zhang et al. reported that prostein expression on circulating tumor cells is an independent predictor of adverse outcomes in mCRPC [96].

Spatial transcriptomics has identified UQCRB and LBH as significant genomic markers with expression levels that progressively increase with higher Gleason scores. Quan et al. validated these findings in the TCGA-PRAD dataset and through immunohistochemistry, with functional assays demonstrating that knockdown of these genes suppresses proliferation and invasion in PCa cell lines [97].

Integrating genomic and phenotypic biomarkers can further improve precision medicine. For example, Davoudi et al. reviewed how germline and somatic genetic testing, as well as polygenic risk scores, can inform treatment decisions in patients with mCRPC. Their work emphasizes that alterations in DNA damage repair genes such as Breast Cancer gene 1 and 2 (BRCA1/2), Ataxia Telangiectasia Mutated (ATM), and Checkpoint Kinase 2 (CHEK2) and aberrations in key signaling pathways, including Androgen Receptor (AR) and the Phosphatase and Tensin Homolog/Phosphoinositide 3-Kinase–Protein Kinase B (PTEN/PI3K-AKT) pathway, not only predict therapeutic response (for example, to Poly(ADP-ribose) polymerase (PARP) inhibitors and Akt inhibitors) but also offer prognostic insights [25].

As summarized by Hatano and Nonomura in their review of PCa genomic profiling, PCa often carries changes such as TMPRSS2–ETS (transmembrane protease, serine 2–E26 transformation-specific) gene fusions and loss of tumor suppressor genes including tensin homolog (PTEN), Retinoblastoma 1 (RB1), and Tumor Protein p53 (TP53), which are linked to tumor progression and worse outcomes. On the other hand, mutations in SPOP define a subgroup of patients that may respond better to androgen receptor-targeted therapy. New approaches like liquid biopsy and broad sequencing panels now make it possible to detect these changes in a less invasive way, helping to capture tumor heterogeneity and treatment resistance. These tools also allow the identification of clinically relevant alterations in DNA repair genes and the PI3K–Akt pathway that can guide the use of targeted therapies [98].

Genomic information can also be combined with imaging. Ferro et al. discussed radiogenomics, the correlation of imaging features from mpMRI with underlying genetic alterations, to improve prognostication. Their review suggests that radiomic signatures, when combined with genomic data, can enhance risk stratification by identifying distinct molecular subtypes and tumor heterogeneity [16].

Moreover, Fenton et al. provide a broader perspective on genomic applications in PCa care, discussing not only the technical advances in genomic profiling but also the challenges of ensuring equitable access to these precision medicine tools. They stress that while genomic markers offer powerful insights for risk stratification and targeted therapy, it is critical that these innovations be implemented in a manner that benefits all patient populations regardless of socioeconomic or ethnic background [99].

Table 2 summarizes the main categories: clinical, biochemical, inflammatory, metabolic, histopathological, and genomic, along with representative examples, applications, and sources.

Table 3 provides a comparative overview of the evidence level, clinical readiness, and added value relative to imaging for each non-imaging biomarker category discussed in this section.

The non-imaging markers described in this section are most useful when read alongside imaging findings rather than in isolation. PSA levels and kinetics inform when and how urgently imaging should be triggered; histopathological grade and genomic classifiers help interpret what equivocal imaging findings mean for a given patient; and several metabolic markers, notably choline, have direct functional imaging equivalents in PET/CT. Current guidelines reflect this integration, recommending that staging and treatment decisions be based on combined clinical, pathological, and imaging data rather than any single modality [2,5].

## 3. Role of MRI in Prostate Cancer Prognosis

MRI is rapidly becoming a central tool in PCa management. However, beyond diagnosis and staging, its prognostic value is increasingly investigated, particularly as clinicians seek more precise and personalized risk stratification strategies.

The introduction of mpMRI has enhanced our ability to predict treatment response, recurrence risk, and overall survival. By refining pretreatment risk stratification, mpMRI-derived anatomical and functional parameters have been incorporated into clinical decision making to distinguish indolent disease from aggressive phenotypes, directly informing the choice between active surveillance and definitive treatment. MRI-derived features offer critical prognostic insights, including indicators of recurrence risk and treatment response [102,103,104,105].

Beyond initial diagnosis, MRI is increasingly recognized for monitoring post-treatment outcomes. Functional imaging techniques such as dynamic contrast-enhanced (DCE) MRI and diffusion-weighted imaging (DWI) have demonstrated high sensitivity in detecting local recurrence after surgery or radiation therapy. Additionally, advancements like whole-body MRI (WB-MRI) and PET/MRI hybrid imaging are enhancing the detection of early metastatic progression, contributing to improved long-term prognosis [106,107].

Recent innovations in MRI acquisition, including 3D T2WI, synthetic high b-value images and apparent diffusion coefficient (ADC) maps derived via deep learning models in DWI, and artificial intelligence (AI)-driven image reconstruction, have substantially reduced scan time while preserving diagnostic fidelity [108]. These advances offer the potential to enhance patient throughput, reduce costs, and improve accessibility, making PCa imaging more efficient without sacrificing clinical reliability; however, widespread adoption remains limited by inter-institutional variability, vendor-dependent implementations, and the absence of standardized acquisition protocols.

Notably, novel functional approaches such as RSI-MRI have also demonstrated superior prognostic value, particularly when integrated with biochemical indices like PHI and PSA density (see Section 2.1.4).

The superior performance of MRI in detecting csPCa compared to traditional methods is well established, with mpMRI demonstrating a sensitivity up to 93% versus 48% for TRUS-guided biopsy [18]. In addition to its role in tumor detection, MRI significantly improves staging accuracy, particularly in identifying extraprostatic extension (EPE) and seminal vesicle invasion (SVI), both critical predictors of adverse oncologic outcomes [106,107]. MRI also contributes to dynamic risk assessment during surveillance. T2-weighted and diffusion-weighted imaging can detect radiologic indicators of disease progression, including increased lesion conspicuity, EPE, and SVI, features that may prompt reclassification to a higher risk category and justify curative intervention [5]. As MRI technology continues to evolve, its integration into prognostic models, coupled with advancements in AI-driven analysis and novel imaging techniques, is expected to further refine personalized treatment strategies and improve patient outcomes.

### 3.1. Multiparametric Whole-Body MRI

Traditional imaging for metastatic PCa staging typically involves a combination of technetium-99m (^99m^Tc) BS and contrast-enhanced CT of the chest, abdomen, and pelvis. While these modalities have long served as the clinical standard, they are limited by low sensitivity for early metastatic disease and suboptimal tissue characterization. Recent advances have positioned WB-MRI as a compelling alternative, offering superior diagnostic performance for both skeletal and nodal metastases [109,110].

WB-MRI is a versatile, non-invasive modality that does not require contrast agents or ionizing radiation. It offers comprehensive whole-body coverage with minimal patient preparation and few contraindications. When integrated with functional sequences, including DWI and relative fat fraction (rFF%), WB-MRI enables sensitive detection of bone marrow metastases and longitudinal assessment of treatment response [110]. This makes it particularly valuable in staging high-risk PCa, evaluating therapy outcomes, and guiding management of BCR.

Multiparametric WB-MRI incorporates several advanced imaging techniques: high-resolution T2-weighted imaging (T2WI) delineates prostatic anatomy; DWI detects cellular density changes associated with malignancy; DCE-MRI assesses tumor vascularity; and magnetic resonance spectroscopic imaging (MRSI) provides metabolic profiling. Though MRSI is less commonly used, its ability to quantify metabolites like choline and citrate may offer additional prognostic insights in selected cases.

In parallel, PSMA PET/CT (prostate-specific membrane antigen) is a powerful modality for detecting metastatic lesions, particularly small-volume lymph node and visceral disease. Comparative studies show strong concordance between WB-MRI and PSMA PET/CT, though the latter offers higher sensitivity for subcentimeter nodal metastases [111].

However, PSMA PET/CT has limitations in patients with low PSMA expression or under ongoing ADT, which may yield false-negative results. WB-MRI, being independent of molecular expression, remains effective in such cases and serves as a robust alternative for disease monitoring in the presence of tumor heterogeneity [109].

Current guidelines continue to recommend CT and BS for metastatic workup, yet these methods face several challenges. BS is an indirect method, detecting osteoblastic activity rather than tumor cells directly, and is prone to false positives due to the FLARE phenomenon (a transient increase in osteoblastic activity on bone scans following effective treatment, which can mimic disease progression) during post-treatment healing [105,112,113]. CT, while effective for detecting lymphadenopathy and visceral metastases, lacks sensitivity for early or diffuse marrow lesions due to the variable appearance of PCa metastases (sclerotic, lytic, or mixed) [113,114].

MRI, and specifically WB-MRI, has shown superior performance in characterizing bone and marrow involvement. Its sensitivity for detecting early skeletal metastases surpasses that of both CT and BS, particularly in patients with low PSA levels. Rydh et al. reported that MRI identified bone metastases in 17% of patients with PSA<20 ng/mL, outperforming BS in early-stage disease detection [115]. More recent meta-analytic evidence confirms the high diagnostic performance of MRI for bone metastasis detection in prostate cancer [116], while contemporary multiparametric WB-MRI further extends this advantage in metastatic disease assessment [105].

Lecouvet et al. demonstrated that WB-MRI outperformed the traditional combination of BS with targeted X-rays (TXR) and contrast-enhanced CT for bone metastasis detection (sensitivity 98–100% vs. 86%), and matched CT for nodal evaluation, in 100 high-risk PCa patients, with comparable specificity across modalities [117]. Pasoglou et al. further validated the use of WB-MRI combined with mpMRI for comprehensive, single-session staging. Their approach yielded 100% sensitivity and specificity for metastatic disease detection and improved staging accuracy, with a 13.6% increase in AUC compared to standard methods (*p* = 0.039) [118].

It is important to note that the true sensitivity of imaging techniques for detecting bone metastases cannot be definitively established, as histopathologic confirmation is rarely feasible. As a result, most studies rely on composite reference standards or longitudinal follow-up, which may bias reported sensitivity estimates toward the most sensitive modality and partly explain reports of near-100% sensitivity. Furthermore, while WB-MRI has shown strong concordance with PSMA PET/CT, direct head-to-head comparative studies would be valuable to more accurately assess the relative performance of these modalities for detecting bone and lymph node metastases [110,117].

### 3.2. MRI in Nodal Disease Prognosis

Accurate nodal staging is essential for risk stratification and optimal treatment planning. While mpMRI is established for local tumor evaluation, its role in nodal prognostication is still limited by suboptimal sensitivity, particularly for micrometastatic disease.

Despite its strengths in tumor localization, mpMRI demonstrates relatively low sensitivity for detecting nodal metastases. As reviewed by Lebastchi et al., MRI sensitivity for nodal metastasis is approximately 39% [119]. These findings highlight the risk of nodal under-staging in clinically node-negative patients.

The prognostic relevance of advanced mpMRI-derived parameters is supported by several recent studies. For example,

- **ADC** values from DWI have been inversely correlated with tumor aggressiveness, as reflected in Gleason score [120].
- **Radiomics-based ML models** leveraging texture and shape features from mpMRI have demonstrated predictive power for lymph node metastasis and oncologic outcomes [121,122,123].

Given the limitations of conventional mpMRI for nodal staging, hybrid PET/MRI—particularly using PSMA-targeted tracers—offers superior accuracy by integrating molecular and anatomical insights [124].

However, mpMRI’s prognostic reliability remains constrained by its inability to detect micrometastases. Integration of imaging biomarkers with clinical and genomic data into AI-driven nomograms could enhance prognostic precision.

### 3.3. MRI Biomarkers in Prostate Cancer Prognosis

MRI-derived biomarkers have shown potential for the prognosis of PCa by reflecting tumor aggressiveness, recurrence likelihood, and metastatic potential. These biomarkers, both quantitative and qualitative, can serve as inputs for risk stratification, therapy planning, and post-treatment surveillance.

Among the most clinically relevant MRI-derived biomarkers are the following:

- **PI-RADS score**, initially introduced as version 1 in 2012 by the European Society of Urogenital Radiology (ESUR) to standardize prostate MRI interpretation [125], and later updated to version 2 in 2015 and version 2.1 in 2019 [20,126]. Higher PI-RADS scores are associated with clinically significant PCa and increased risk of BCR [127].
- **ADC** values, where lower ADC is indicative of increased cellularity and inversely correlates with Gleason score [120]. Lower pretreatment ADC has been shown to independently predict BCR after radical prostatectomy [128], although a meta-analysis restricted to radiotherapy cohorts did not confirm a significant pooled association [129].
- **Lesion volume and tumor burden**, which are associated with adverse pathology such as SVI and nodal metastasis [129], and independently predict biochemical recurrence after radiotherapy [129,130].
- **EPE** and **SVI**, both of which signify locally advanced disease and are strong predictors of early recurrence and lower cancer-specific survival [106,107].
- **Radiomics-based features** extracted through high-throughput image analysis augment conventional clinicopathologic metrics (PSA, Gleason score, PI-RADS) and improve prediction of BCR after definitive treatment [131,132].

A summary of the most established MRI biomarkers and their clinical implications is presented in Table 4.

### 3.4. MRI in Predicting Treatment Response and Recurrence

MRI has been used for assessing local treatment response and recurrence in PCa. The mpMRI, which includes T2WI, DWI, and DCE MRI, has proven effective in evaluating post-treatment changes and predicting BCR. Recent studies have further used radiomics and AI to enhance MRI’s prognostic capabilities.

Several MRI-derived biomarkers have demonstrated potential in predicting earlier treatment recurrence. Diffusion-weighted imaging, particularly using ADC values, has been correlated with tumor cell density and recurrence. Effective therapy, particularly following radiotherapy, is often associated with an early increase in ADC values reflecting loss of cellular integrity and decreased tumor cellularity [133,134]. In the neoadjuvant setting, serial mpMRI has also demonstrated value: baseline tumor burden on MRI was identified as an independent predictor of minimal residual disease in high-risk PCa patients undergoing neoadjuvant enzalutamide plus ADT prior to surgery [135].

Biochemical recurrence following treatment remains a major prognostic challenge, with 20–40% of patients experiencing BCR within 10 years after RP, and 30–50% after radiation therapy [132]. Studies have shown that radiomics-based MRI analysis can improve BCR risk prediction. Shiradkar et al. developed a machine learning model using radiomic features from biparametric MRI (bp-MRI, T2WI + ADC maps), achieving an AUC of 0.73 on an independent validation-set in predicting BCR. Their findings indicated that radiomic texture features, such as Haralick and CoLlAGe features, provided superior prognostic accuracy compared to PSA, ISUP GG, and PI-RADS v2 [132].

Similarly, Dinis Fernandes et al. explored the use of T2-weighted MRI-based radiomics to predict five-year BCR in high-risk PCa patients treated with EBRT. Their model, based on whole-prostate imaging features, achieved an AUC of 0.63, outperforming standard clinical features (AUC = 0.51) [136].

Following surgery or radiation therapy, detecting local recurrence is critical for guiding salvage treatments. Panebianco et al. introduced the Prostate Magnetic Resonance Imaging for Local Recurrence Reporting (PI-RR) criteria, providing a standardized approach for identifying recurrence on post-treatment MRI. The use of DCE-MRI and DWI has proven effective in differentiating recurrent tumors from post-treatment fibrosis [137].

### 3.5. Advancements in MRI Technology for Prognosis

Recent advances in AI and ML have substantially enhanced MRI’s prognostic capabilities. Radiomics-based classifiers, such as the one developed by Shiradkar et al., outperformed traditional clinical predictors for BCR prediction, showing that deep learning models integrating quantitative imaging features can improve risk stratification [132]. Similarly, Karzai et al. reported that low baseline mpMRI relative tumor burden was the strongest predictor of pathologic response (AUC 0.89, cutoff 8.1%) in high-risk PCa patients receiving neoadjuvant enzalutamide plus ADT, highlighting the role of pretreatment imaging biomarkers in guiding therapy decisions [135].

Deep-learning-based reconstruction techniques, including compressed sensing approaches, have improved image quality while reducing scan times, enabling faster and more accurate imaging without compromising diagnostic integrity [138]. For example, deep-learning-enhanced T2WI increased sharpness and diagnostic confidence in PCa detection while halving acquisition times [139]. By enhancing signal-to-noise ratios (SNRs) and optimizing contrast-to-noise performance, these methods improve lesion conspicuity and inter-reader agreement in PI-RADS scoring [140], which may, in turn, support more reliable risk stratification in prognostic workflows.

Radiomics has, likewise, shown strong potential for extracting high-dimensional imaging biomarkers that correlate with disease progression and treatment response. AI-driven radiomics pipelines facilitate the identification of subtle imaging patterns that are often imperceptible to human observers [141,142]. Beyond BCR prediction, these models have also demonstrated strong predictive performance for progression-free survival (PFS) [143], with hybrid models combining radiomic features and clinical parameters achieving superior prognostic accuracy over radiomics alone. By leveraging quantitative imaging features, radiomics can also support differentiation between aggressive and indolent PCa [142,144].

These findings are encouraging, but the clinical translation of AI-driven radiomics in MRI remains constrained by important methodological factors. Most reported models were trained and tested on retrospective, single-center datasets with limited sample sizes, and none has yet received regulatory approval or been endorsed in clinical guidelines for routine prognostic use [145]. Performance metrics such as AUC values, obtained under these controlled conditions, are unlikely to generalize across institutions without independent external validation [146]. Until prospective, multicenter studies confirm their reproducibility and added clinical value over established prognostic tools [20], these approaches should be regarded as research instruments rather than practice-ready applications.

### 3.6. Limitations and Future Directions

Despite substantial progress, several challenges limit the routine clinical adoption of advanced MRI for prognosis.

A major limitation is the lack of standardization in imaging protocols and interpretation criteria. Variability in acquisition parameters and differences in reporting practices across institutions can lead to inconsistencies in diagnosis and risk stratification [145]. Accessibility further complicates implementation, as techniques such as mpMRI and hybrid imaging are not uniformly available across health systems, with infrastructure and trained personnel for advanced prostate imaging remaining particularly scarce in low- and middle-income countries [147]. Moreover, even with high sensitivity, MRI may yield false-negative findings in low-grade or small-volume tumors, necessitating complementary diagnostic tools [102].

Hybrid imaging approaches, most notably PSMA PET/MRI, integrate molecular and anatomical information and have shown improved performance compared to mpMRI alone in early studies [148]. However, such hybrid modalities may exacerbate accessibility issues due to their high cost and limited availability [149,150], highlighting the importance of balancing diagnostic benefit with real-world feasibility.

AI-based MRI techniques face significant challenges in terms of standardization, interpretability, and generalizability, which limit their immediate clinical adoption. Current models are often optimized for specific institutional protocols, restricting reproducibility across centers and posing barriers to widespread clinical translation. In addition, most MRI radiomics approaches are applied offline and cannot yet be exploited in real time during localized treatments such as radiotherapy or surgery, limiting their direct impact on intra-procedural decision making. Nevertheless, AI-driven image analysis and radiomics pipelines hold promise for mitigating variability by automating segmentation, enhancing lesion characterization, and improving reproducibility [145,151].

Emerging solutions, including MRI–ultrasound fusion systems and hybrid imaging platforms, may enable the integration of MRI-derived radiomic features into treatment workflows. If combined with advanced AI frameworks and validated on sufficiently diverse datasets, these approaches could reduce inter-reader variability, support real-time guidance, and strengthen the robustness of prognostic and predictive models. Similarly, integrating multimodal imaging data, such as PET/MRI fusion [152], and incorporating molecular MRI biomarkers (e.g., DCE-MRI, DWI) into AI-enhanced frameworks may further refine risk stratification and support precision oncology applications [102].

Overall, future efforts should prioritize harmonizing imaging standards, expanding access to advanced modalities, and validating AI-enhanced frameworks in large, multi-institutional cohorts to fully realize the prognostic potential of MRI in PCa.

A comparative summary of the key imaging modalities discussed in this chapter, including their prognostic utilities, strengths, and limitations, is provided in Table 5.

Table 6 provides a concise comparative overview of the evidence level and clinical readiness for each of the modalities discussed in this section.

## 4. Role of CT, PET, and Their Hybrid Modalities in Prostate Cancer Prognosis

In recent years, hybrid imaging modalities, particularly PET/CT and PET/MRI, have increasingly been used for the prognosis of PCa. By combining the anatomical precision of CT or MRI with the molecular specificity of PET, these modalities offer a comprehensive understanding of disease burden, aggressiveness, and progression patterns [9,55,157,158].

### 4.1. CT-Based Prognostic Markers

CT on its own has limited value in detecting PCa, but it can provide useful prognostic information. For example, CT-based measures of body composition have been linked to clinical outcomes in mCRPC: high subcutaneous adipose tissue (SAT) is independently associated with longer overall survival [159], sarcopenia (low skeletal muscle mass, SMM) predicts radiographic progression and shorter overall survival [160], and elevated subcutaneous and visceral adipose tissue (VAT) are associated with improved response to androgen-signaling inhibitors [161]. More recently, deep-learning whole-body CT segmentation has enabled automated quantification of these compartments, with low SAT and high intermuscular adipose tissue (IMAT) ratios independently predicting poorer outcomes in mCRPC patients undergoing ^177^Lu-PSMA radioligand therapy [162]. Beyond body composition, conventional CT remains the standard modality for detecting visceral metastases in mCRPC, which are themselves an independent negative prognostic factor [159]. Cone beam CT (CBCT) radiomics has also shown potential in predicting staging, ISUP GG, PSA levels, and risk of BCR, particularly during EBRT [163,164].

### 4.2. PET Tracers and Their Prognostic Value

Different PET tracers are used in PCa prognosis:

#### 4.2.1. FDG PET/CT

Although FDG PET/CT is not the preferred imaging modality for staging most prostate cancer patients, it becomes useful when the disease is aggressive, poorly differentiated, or progressing despite therapy. In these settings, FDG uptake reflects higher glucose metabolism, which often corresponds with faster growth and worse outcomes [165].

In localized very-high-risk disease, intraprostatic FDG uptake can already carry prognostic information. Lavallée et al. studied patients with localized Gleason 8–10 prostate cancer and showed that higher FDG uptake identified a subgroup with early recurrence and earlier resistance to castration, supporting the idea that FDG can reveal aggressive biology even before widespread metastases [166]. In advanced metastatic disease, FDG PET/CT is more consistently prognostic. Jadvar et al. reported that baseline FDG PET/CT metrics in mCRPC were associated with overall survival, supporting the role of FDG as a whole-body marker of aggressive tumor burden [167]. In a multicenter cohort, Bauckneht et al. found that FDG-derived volumetric measures, especially metabolic tumor volume, provided independent prognostic value and could also relate to how long patients benefit from systemic treatment [168]. FDG PET/CT is also helpful when prostate cancer shifts toward neuroendocrine features, where PSMA expression can decrease and FDG uptake can increase. In neuroendocrine prostate cancer, Shen et al. showed that higher primary-tumor SUV_max_ was associated with worse overall survival, and FDG uptake helped distinguish histopathologic subtypes [169]. An important clinical role of FDG PET/CT in mCRPC is to detect discordant disease, meaning lesions that are FDG-avid but PSMA-negative. These lesions often represent dedifferentiation and are linked to poorer outcomes and reduced suitability for PSMA-targeted radioligand therapy. The TheraP trial required both PSMA PET and FDG PET and excluded patients with FDG-positive/PSMA-negative sites, highlighting the clinical relevance of discordance for treatment selection [21]. In contrast, the VISION trial used PSMA PET selection without mandatory FDG PET, and still showed survival benefit from ^177^Lu-PSMA-617 in eligible patients [22]. More recently, Demirci et al. evaluated VISION-eligible patients who received ^177^Lu-PSMA-617 and showed that FDG-avid discordant disease was associated with lower response and shorter survival, suggesting that discordance is a strong negative prognostic factor even among patients treated with radioligand therapy [170]. A prospective Canadian cohort using a triple-tracer strategy also reported a high prevalence of FDG-positive/PSMA-negative lesions and linked heterogeneity to shorter overall survival [171].

#### 4.2.2. Choline PET/CT

^11^C-choline PET/CT has been used for recurrence imaging, detecting pelvic and extra-pelvic disease and guiding treatment [80]. It has also been applied to plan extended nodal radiotherapy, with improved outcomes reported in selected cohorts [172]. However, in most contemporary settings, choline tracers have largely been superseded by PSMA-targeted PET for staging and recurrence evaluation, and their use is mainly limited to situations where PSMA PET is unavailable.

#### 4.2.3. PSMA PET/CT and PSMA PET/MRI

PSMA-targeted PET imaging has transformed PCa management by providing high sensitivity for metastatic disease and substantially improving staging accuracy compared with conventional imaging, frequently leading to changes in clinical management [12,173,174,175]. Beyond lesion detection, PSMA PET enables quantitative assessment of whole-body tumor burden, which has become increasingly relevant for prognosis. Volumetric parameters such as PSMA tumor volume (PSMA-TV) and total lesion PSMA (TL-PSMA) correlate with metastatic extent [176], and PSMA PET-derived metrics more broadly have demonstrated prognostic value across disease stages, including BCR and metastatic disease [177]. PSMA PET/MRI combines molecular sensitivity with multiparametric soft-tissue assessment and has shown improved specificity for clinically significant PCa compared to mpMRI alone in early studies, particularly in equivocal PI-RADS 3 lesions [148].

Studies using ^18^F-labeled tracers, such as ^18^F-PSMA-1007 and [^18^F]DCFPyL, have further supported the prognostic relevance of PSMA PET-derived metrics. Higher PSMA-TV, TL-PSMA, and SUVmax values have been associated with worse PFS and earlier disease progression, highlighting the added value of quantitative PSMA PET beyond visual interpretation alone [178,179]. Several recent analyses have evaluated combined volumetric and dissemination features derived from PSMA PET, such as whole-body total lesion PSMA burden (PSMA-TTL) and maximum dissemination distance (Dmax), suggesting that global tumor burden measures provide independent prognostic stratification beyond conventional clinicopathological factors in selected patient populations [177,179].

Despite these advances, important limitations remain. Low or heterogeneous PSMA expression can result in false-negative findings, particularly in dedifferentiated or aggressive tumor phenotypes, while benign uptake in structures such as ganglia, fractures, or inflammatory processes may lead to false-positive interpretations [180]. In addition, small-volume or micrometastatic disease may fall below the spatial resolution of PET imaging [173,181]. Variability in tracer properties, image acquisition protocols, segmentation methods, and reporting standards continues to challenge harmonization of quantitative PSMA PET biomarkers across centers and studies [182]. Nevertheless, the integration of PSMA PET-derived quantitative features into prognostic and AI-based models represents a major step toward personalized risk assessment in prostate cancer.

### 4.3. Radiomics and Artificial Intelligence

Radiomics and AI have added prognostic value to PET imaging in prostate cancer by enabling objective quantitative analysis [183,184]. Central to these approaches is tumor segmentation, as most PET-derived biomarkers, including molecular tumor volume (MTV), total lesion activity (TLA), and radiomics features, depend directly on lesion delineation [185]. Manual segmentation is time-consuming and subject to inter-observer variability, which limits reproducibility and scalability, particularly in whole-body imaging [146].

To address these limitations, deep-learning-based methods, predominantly convolutional neural networks, now enable automated tumor segmentation on PSMA and FDG PET/CT, improving reproducibility for extracting volumetric biomarkers such as MTV and TLA across applications from intraprostatic tumor delineation to whole-body metastatic disease assessment [186,187]. These automated metrics have, in turn, been used to build prognostic models: Leung et al. applied deep-learning-based segmentation on ^18^F-DCFPyL PSMA PET/CT to compute whole-body MTV and TLA and derived a risk model that significantly stratified follow-up PSA levels in advanced prostate cancer [188], while Qiao et al. showed that FDG PET/CT radiomics features—including tumor-to-liver ratio (TLR) and composite radscores—were associated with BCR and response to ADT [184].

Beyond single-modality analysis, AI-based segmentation enables the integration of imaging biomarkers with clinical variables in multivariate prognostic models. Larose et al. developed a Bayesian Sequential Network combining FDG PET/CT-derived features with clinical data, demonstrating improved prediction of LNI and BCR-free survival, particularly through multimodal integration, compared with clinical-only approaches such as CAPRA and MSKCC, while baseline nomograms remained superior for prostate-cancer-specific survival [189]. Overall, by improving reproducibility and enabling large-scale radiomics and multimodal modeling, AI-driven segmentation can support more robust risk stratification and facilitate the clinical translation of quantitative PET biomarkers in prostate cancer.

Two related extensions of PET radiomics are gaining attention in prostate cancer. The first is theranostic-oriented: PSMA PET radiomics has been studied as a way to predict response to ^177^Lu-PSMA-617 radioligand therapy, with image features capturing spatial heterogeneity that may reflect dosimetric and biological variability in treatment outcome [183]. The second is radiogenomics—using radiomic features to probe the genomic and molecular basis of imaging phenotypes. Most of the prostate cancer evidence here has come from mpMRI rather than PET, but the same principles are beginning to be applied to PSMA imaging [190]. Both areas remain investigational. There are no prospective multicenter validations to date, and neither has been incorporated into clinical guidelines. Single-center retrospective performance figures should, therefore, be read as hypothesis-generating, not as evidence ready for routine use.

### 4.4. Clinical and Prognostic Impact

In clinically node-positive prostate cancer, PSMA PET staging has been associated with improved biochemical failure-free and overall survival compared with conventional staging [191]; even a negative PSMA PET/CT carries prognostic value in the setting of biochemical recurrence [56]. The PROMISE framework integrates PSMA PET/CT findings into the molecular imaging Tumor-Node-Metastasis (TNM) staging system, referred to as miTNM, to support decision making [192]. During ^177^Lu–PSMA-617 therapy, SPECT/CT-derived total tumor volume and the appearance of new lesions identify higher-risk patients and predict outcomes [193].

A comparative summary of key prognostic imaging modalities and radiotracers used in PCa is presented in Table 7, highlighting their respective strengths and limitations.

Table 8 summarizes the evidence level and clinical readiness of the PET tracers and hybrid modalities discussed in this section.

## 5. Role of Ultrasound in Prostate Cancer Prognosis

Ultrasound is most commonly used for prostate biopsies, allowing precise sampling of suspicious areas to detect cancer, particularly in the peripheral zone. Beyond diagnosis, TRUS can play a role in treatment guidance, such as brachytherapy, cryotherapy and high-intensity focused ultrasound (HIFU), enabling precise targeting of cancerous tissue. Additionally, advanced TRUS techniques, including contrast-enhanced ultrasound (CEUS) and elastography, improve sensitivity by highlighting tumor vascularity and stiffness. While traditional TRUS has limitations in distinguishing cancer from benign conditions, its integration with MRI in fusion techniques has significantly enhanced its accuracy. TRUS is an essential tool in PCa care, providing valuable insights for diagnosis, staging, and personalized treatment planning [13]. The procedure typically takes 10 to 30 min and is performed on an outpatient basis. Although it may cause temporary discomfort, TRUS is generally regarded as a minimally invasive and low-risk procedure.

A data-driven approach using TRUS has shown promise in improving the diagnostic evaluation of high-grade PCa. By combining TRUS imaging data with clinical variables such as PSA and total prostate volume (TPV), high-grade PCa was identified with an AUC of 0.835 (p=0.007), significantly enhancing the accuracy of pathological cancer grading. Although primarily diagnostic, this method has prognostic implications, as high-grade PCa are strongly associated with aggressive disease and poorer outcomes. The integration of deep learning techniques could further reduce inter-observer variability [198].

TRUS-guided biopsy has demonstrated prognostic value in assessing survival outcomes for elderly PCa patients. Using TRUS biopsy data, a nomogram achieved an AUC of 0.85 for predicting survival in patients living < 4 years, with significant predictors including PSA levels (>50 ng/mL), Gleason score 8–10 (ISUP GG 4–5), and metastases [199].

Novel techniques such as CEUS and elastography have shown significant potential in improving the detection of csPCa. CEUS visualizes the increased microvessel density associated with prostate tumors, while elastography measures tissue stiffness, both of which enhance diagnostic and staging accuracy [14,200,201].

Quantitative CEUS (qCEUS) integrates parameters such as wash-out area under the curve (WoAUC) and rise time with clinical features like PSA density and PI-RADS scores. A predictive model incorporating these factors achieved an AUC of 0.910 in the training cohort and 0.879 in validation, significantly improving risk stratification and reducing unnecessary biopsies [201]. Beyond initial risk stratification, CEUS-derived time–intensity curve (TIC) parameters—peak intensity (PI) and time-to-peak (TTP)—have shown independent prognostic value for post-treatment BCR. Mei et al. combined PI and TTP with established clinical risk factors (lymph node metastasis, Gleason score, pretreatment PSA, and treatment method), with PI and TTP retained as independent multivariate predictors and the integrated model achieving an AUC of 0.953 and sensitivity and specificity exceeding 90% [202].

Micro-ultrasound (microUS) is a high-resolution ultrasound technology introduced as a low-cost alternative to mpMRI for imaging PCa. It employs a 29 MHz transducer, allowing for a superior axial resolution of 70 µm in proximal regions of the prostate, which enables the visualization of prostate features and tumors not resolvable by conventional ultrasound. In the multicenter randomized OPTIMUM trial, Kinnaird et al. demonstrated that microUS-guided biopsy was non-inferior to MRI fusion-guided biopsy for the detection of clinically significant prostate cancer (ISUP GG ≥ 2), with comparable detection rates and without the need for MRI image registration [203]. Earlier comparative studies summarized by Klotz reported similar diagnostic performance between microUS and mpMRI, with microUS showing comparable sensitivity and, in some studies, improved assessment of tumor extent, but slightly lower specificity [204]. Consistent with these findings, Pensa et al. reported that microUS achieved higher index lesion detection compared with retrospective MRI review (91.7% vs. 80%) and improved assessment of tumor extent (52.5% vs. 36.7%), supporting its role as a cost-effective and accessible imaging modality [205].

Shear wave elastography (SWE) provides significant prognostic insights into PCa by quantifying tissue stiffness. Tissue modulus values measured via SWE strongly correlate with tumor grade (r=0.74,p<0.001), with more aggressive cancers exhibiting higher stiffness. Additionally, second-harmonic generation (SHG) imaging revealed changes in collagen orientation and crosslinking around cancerous tissues, with a very strong correlation between collagen orientation and histopathological grade ($r_{s}=0.93,p<0.001$) [206].

Sonazoid-enhanced ultrasound (SEU) has been investigated primarily in the context of prostate cancer detection, where perflubutane-microbubble-enhanced CEUS has demonstrated improved lesion visualization and cancer detection rates compared to systematic biopsy [207]. Preliminary conference data suggest that SEU positivity may also carry prognostic value, with one abstract reporting a significant association between SEU positivity and BCR-FS after radical prostatectomy [208]. However, these findings have not yet been published in a peer-reviewed journal, and the prognostic utility of SEU remains to be validated in prospective studies.

The integration of CEUS and MRI radiomics has shown strong potential for prognostic stratification in PCa. For preoperative risk stratification, combining bp-MRI habitat features, which capture intratumoral heterogeneity by clustering MRI voxels with similar characteristics, together with CEUS radiomics features provided a robust tool for predicting high-risk PCa (Gleason ≥ 8). This combined model achieved AUCs of 0.875 and 0.842 in training and testing cohorts, respectively, outperforming individual MRI-habitat or CEUS-intra models [209].

Finally, ultrasound phenotypic features coupled with proteomics have shown potential in metastatic risk assessment. Features such as lesion length, gland demarcation, blood flow, and lesion state correlated strongly with metastatic biomarkers like heterogeneous nuclear ribonucleoprotein C (HNRNPC), achieving an AUC of 0.904 (p<0.0001). This approach emphasizes the utility of integrating imaging phenotypes with molecular data to enhance risk stratification and prognosis in PCa [210].

A comparative summary of ultrasound modalities and their prognostic applications in PCa is presented in Table 9.

## 6. Conclusions

The integration of multimodal imaging and non-imaging data is an important step toward PCa prognosis. This review shows that combining advanced imaging modalities, including mpMRI, PSMA PET/CT, and newer ultrasound techniques such as CEUS and elastography, with clinical, biochemical, metabolic, histopathological, and genomic biomarkers could refine prognostic models and support more individualized decision making. Developments in radiomics and AI further suggest that subtle imaging and molecular patterns could support the development of predictive models for outcomes such as BCR and PFS. In addition, developing approaches such as multimodal fusion for predicting progression to castration-resistant disease [17], radiogenomics linking imaging to molecular alterations [211], and digital twin frameworks could improve prognostic accuracy and help guide clinical management [212].

**Figure 2:**
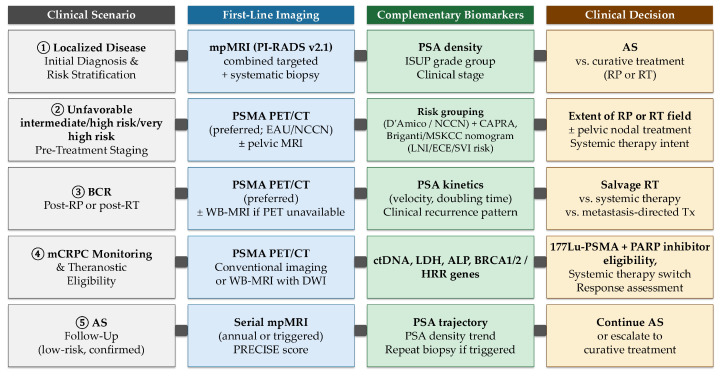
Clinical decision framework for biomarker and imaging tool selection across prostate cancer disease stages. Each lane maps a clinical scenario to the recommended first-line imaging modality, complementary biomarkers, and resulting clinical action. Lane 1 (initial diagnosis): mpMRI/PI-RADS v2.1 with combined targeted + systematic biopsy. Lane 2 (high-risk staging): PSMA PET/CT preferred per EAU/NCCN. Lane 3 (BCR): PSMA PET/CT first-line, WB-MRI when unavailable. Lane 4 (mCRPC and theranostic eligibility): PSMA PET/CT for ^177^Lu-PSMA-617 selection, with conventional imaging or WB-MRI when appropriate; HRR alterations for PARP eligibility. Lane 5 (active surveillance): serial mpMRI with PSA monitoring. Where PSMA PET/CT is unavailable, CT and ^99m^Tc bone scintigraphy remain the fallback. Supporting references for each lane are provided in the corresponding sections of the manuscript; abbreviations are defined in the manuscript abbreviations list.

Translating these findings into practice requires matching the clinical question to the appropriate tool. For initial risk stratification in localized disease, mpMRI guided by PI-RADS v2.1 criteria, combined with targeted biopsy, remains the standard entry point, reducing grade underestimation compared with systematic biopsy alone [18,19,20]. In men under active surveillance, serial mpMRI provides the most reliable non-invasive means of monitoring disease trajectory, though biopsy remains necessary when imaging suggests progression [104,213]. For high-risk primary staging and biochemical recurrence evaluation, PSMA PET/CT is now endorsed by both EAU and NCCN guidelines as the preferred functional imaging modality, given its superior sensitivity at low PSA values and its direct impact on treatment intent [23,66,182,191]. In patients with metastatic castration-resistant disease where PET access is limited, WB-MRI with DWI offers a validated radiation-free alternative to conventional imaging for whole-body burden assessment [110,117]. For theranostic eligibility evaluation, PSMA PET/CT is required to confirm target expression prior to ^177^Lu–PSMA-617 therapy [22,66]. Radiomics and AI-derived metrics should not yet substitute for these guideline-recommended workups; their application remains appropriate in the context of prospective validation studies or institutional research protocols [145,146]. Figure 2 summarizes these decision points as a practical reference for clinical use.

Despite this progress, several challenges remain. A lack of standardization in imaging acquisition, reporting criteria, biomarker assays and high-quality long-term clinical databases continues to generate variability and limits the reproducibility of prognostic tools across institutions. Barriers such as limited interoperability, accessibility issues, and the high cost of advanced imaging and genomic testing may also restrict their widespread clinical use and contribute to healthcare disparities. Furthermore, AI-based methods require careful evaluation to ensure interpretability, transparency, and reproducibility across diverse patient populations [214], while phenomena such as the “Will Rogers effect” (whereby reclassification of patients to a more advanced stage by sensitive imaging can artifactually improve stage-specific survival statistics without genuine clinical benefit) highlight the importance of critically assessing whether reclassification genuinely improves patient outcomes [2].

To advance the field, future research should focus on harmonizing imaging and biomarker standards, conducting prospective multicenter validation studies, and developing frameworks for data governance that ensure interoperability and equitable access. Concrete steps toward standardization include the adoption of Image Biomarker Standardisation Initiative (IBSI)-compliant radiomic feature extraction pipelines to reduce inter-software variability [146], consistent use of structured reporting frameworks such as PI-RADS v2.1, the PSMA Reporting and Data System (PSMA-RADS), and miTNM across institutions [20,182,192], and investment in federated learning architectures that enable multi-institutional AI model development without requiring raw patient data sharing [215]. Open imaging repositories and phantom-based scanner harmonization studies would further reduce acquisition-level variability and support reproducible biomarker research across diverse health systems.

Beyond these infrastructure priorities, each imaging domain presents distinct and addressable research frontiers. For mpMRI, prospective evaluation of abbreviated biparametric protocols in active surveillance cohorts would address cost and accessibility barriers that currently limit serial monitoring in resource-constrained settings [104,213]. For PSMA PET/CT, trials are needed to define optimal imaging timepoints during systemic therapies—including androgen receptor pathway inhibitors—and to validate ^18^F-labeled PSMA tracers as logistically accessible alternatives to ^68^Ga-PSMA in centers without on-site cyclotrons [66,182]. In theranostics, linking quantitative PSMA PET features such as expression heterogeneity and total tumor volume to dosimetric response prediction for ^177^Lu–PSMA-617 represents a high-priority translational frontier [22]. For ultrasound, integration of microUS platforms with MRI-fusion targeting and AI-guided lesion identification could improve biopsy yield in patients with prior negative MRI, a population currently lacking a reliable next-line tool [205]. In the radiogenomics domain, prospective studies correlating imaging phenotypes with actionable somatic alterations such as *BRCA2* and *CDK12* mutations could enable non-invasive triage for genomic testing and downstream PARP-inhibitor patient selection [216]. Finally, developing foundation models trained on curated multimodal PCa datasets and evaluated under regulatory-grade explainability frameworks represents a necessary step before AI-derived prognostic scores can be responsibly integrated into clinical practice [215]. By addressing these limitations, the integration of imaging and non-imaging biomarkers could become central to precision oncology in PCa, facilitating reliable prognostic assessment and improving patient outcomes.

## Figures and Tables

**Table 1:** Summary of risk stratification tools for prostate cancer.

Tool	Risk Categories	Criteria Used	Strengths/Notes	Ref.
D’Amico	Low/intermediate/high	PSA, Gleason score (corresponding to ISUP GG), clinical stage	Simple and widely used; limited granularity	[32]
NICE	3-tier	PSA, T stage, ISUP GG, % cores	UK guideline-based; treatment-oriented	[33]
EAU	Low to very high	PSA, ISUP GG, clinical stage, biopsy information	Widely used in Europe	[5]
AUA	Low/Favorable Intermediate/Unfavorable Intermediate/High	PSA, ISUP, clinical stage, % biopsy cores positive	Subcategorizes intermediate risk; no “very low” or “very high” categories	[34]
NCCN	6-tier	PSA, ISUP GG, T stage, % cores	Includes favorable/unfavorable intermediate risk	[23]
MSKCC	Continuous score	PSA, age, ISUP GG, stage, biopsy info	Personalized recurrence prediction	[35]
CAPRA	0–10 scale	Age, PSA, ISUP GG, stage, biopsy data	Simple and validated	[36]
CPG	5 groups	PSA, stage, ISUP GG	High accuracy; validated nationally	[37,38]

Category labels differ slightly across guidelines; tier counts reflect major prognostic groupings used for clinical decision making.

**Table 2:** Summary of prognostic marker categories (excluding imaging modalities) in prostate cancer.

Category	Details
**Clinical**	**Examples:** Performance status, pain, digital rectal examination (DRE), age **Applications:** Risk stratification, treatment selection **Sample Source:** Questionnaire and physical examination **References:** [26,32,68,100]
**Biochemical**	**Examples:** LDH, ALP, Fib, PSA, hemoglobin **Applications:** BCR prediction, therapy monitoring, prognostication **Sample Source:** Blood **References:** [26,59]
**Inflammatory**	**Examples:** CRP, NLR **Applications:** Prognosis, aggressiveness, survival prediction **Sample Source:** Blood **References:** [61,62]
**Metabolic**	**Examples:** Sarcosine, choline, glutamate **Applications:** Early detection, aggressiveness prediction **Sample Source:** Urine/plasma **References:** [73,78]
**Histopathological**	**Examples:** IDC-P, Cribriform morphology, GGs (ISUP grade) **Applications:** Recurrence prediction, survival stratification **Sample Source:** Tissue (biopsy/surgery) **References:** [84,88]
**Genomic**	**Examples:** Decipher, ctDNA fraction, PCDI, homologous recombination repair gene mutations (HRRm) **Applications:** Molecular stratification, therapy response prediction **Sample Source:** Tissue/liquid biopsy **References:** [25,53,94,95]

**Table 3:** Comparative overview of non-imaging biomarkers in prostate cancer prognosis.

Biomarker Category	Evidence Level	Clinical Readiness	Added Value vs. Imaging Alone
PSA-based Markers	Guideline-endorsed; synthesized across major society guidelines [44,53]	Standard of care [5,66]	Longitudinal disease monitoring between imaging timepoints; cost-effective screening trigger [66]
Clinical Risk Scores	Guideline-endorsed; large multicenter registry studies [38]	Standard of care [5,66]	Provides treatment-decision framework that contextualizes imaging findings; predicts mortality beyond imaging stage [5,66]
Inflammatory/Coagulation Markers	Largely validated; prospective cohorts and meta-analyses [26,61]	Emerging [61,62]	Captures systemic inflammatory burden not visible on imaging; improves mCRPC survival stratification [26]
Metabolic Markers	Investigational; predominantly single-center retrospective studies [71,78]	Research only [71]	May identify imaging-occult aggressive biology through metabolic phenotyping [71,78]
Histopathological Grading	Guideline-endorsed; large population-based registry studies [84,88]	Standard of care [5,66]	Gold standard for tumor aggressiveness; imaging cannot replace tissue diagnosis for Gleason grading [18,84]
Genomic Classifiers	Commercially available; prospective-retrospective validation [53]	Emerging—guideline-supported in select settings [66]	Adds molecular risk stratification beyond clinical and imaging parameters; refines treatment decisions in equivocal imaging settings [66]
Liquid Biopsy (ctDNA/cfDNA)	Emerging; prospective cohort validation [94]	Research/selected centers [42,94]	Real-time systemic tumor monitoring between imaging timepoints; detects resistance mechanisms not captured by imaging [94,101]

Evidence Level: Guideline-endorsed = supported by large prospective or registry studies in major guidelines; Largely validated = prospective or large retrospective multicenter data; Investigational = predominantly single-center or pilot data. Clinical Readiness: Standard of care = guideline-mandated; Emerging = available but adoption varies; Research/selected centers = not yet in routine clinical guidelines. cfDNA = cell-free DNA.

**Table 4:** Summary of MRI biomarkers and their prognostic implications in prostate cancer.

MRI Biomarker	Prognostic Insight	Associated Outcome	References
PI-RADS Score	Higher PI-RADS scores are linked to more aggressive disease	Increased risk of BCR, shorter recurrence-free survival (RFS)	[127]
ADC	Lower ADC values indicate higher tumor cellularity	Independent predictor of BCR after radical prostatectomy	[120,128]
Lesion Volume	Larger tumor volume correlates with adverse pathology	Associated with higher risk of BCR, SVI, LNI	[129,130]
Index Lesion Burden	High lesion-to-prostate volume ratio is prognostic of recurrence	Early BCR post-treatment	[127]
EPE	Suggests local tumor spread beyond prostate capsule	Predictor of poor oncologic outcomes	[106]
SVI	Indicates locally advanced disease	Associated with recurrence and lower survival rates	[107]
Radiomics-based Features	High-dimensional texture features capture tumor heterogeneity	Improved prediction of BCR, outperforming conventional clinicopathologic metrics	[131,132]

**Table 5:** Summary of imaging modalities for prostate cancer prognosis.

Modality	Prognostic Utility and Considerations
**mpMRI**	**Sensitivity:** Moderate–high for bone metastases. **Nodal Detection:** Limited–moderate [119]. **Strengths:** Multiparametric data (T2WI, DWI, DCE), no radiation, high soft tissue contrast. **Limitations:** Poor micrometastasis detection, operator dependence, and low specificity for lymph node metastases. **Clinical Use:** Tumor localization, active surveillance, lesion characterization [103].
**WB-MRI**	**Sensitivity:** High for bone metastases in comparative studies [110,115,116,117]. **Nodal Detection:** Moderate–high. **Strengths:** Whole-body view, DWI sensitivity, no contrast or radiation. **Limitations:** Sensitivity estimates depend on non-pathologic reference standards; limited detection of small lymph nodes <8 mm [111]. **Clinical Use:** Staging, treatment response evaluation, BCR [110].
**PSMA PET/CT**	**Sensitivity:** High for bone metastases; moderate for nodal metastases. **Strengths:** Detects PSMA+ lesions across bone, node, and visceral sites; high specificity for metastatic disease. **Limitations:** False negatives in PSMA-low tumors [109]; reduced uptake post-ADT [153]. **Clinical Use:** Recurrence detection; staging of high- and very-high-risk primary PCa.
**BS**	**Sensitivity:** Low–moderate [5,154]. **Strengths:** Widely available, standard for decades. **Limitations:** Indirect osteoblastic activity detection, FLARE effect, poor soft tissue detail [113,155] and substantially lower sensitivity than PSMA PET/CT [12]. **Clinical Use:** Bone metastases screening where advanced imaging is unavailable [2,66].
**CT + Contrast**	**Sensitivity:** Low for bone metastases. **Nodal Detection:** Moderate for enlarged nodes. **Strengths:** Good for visceral metastases, fast, accessible. **Limitations:** Poor detection of early bone metastases, difficulty distinguishing lesion types, and low specificity [114,156]. **Clinical Use:** Routine staging when PSMA PET or MRI are unavailable [66].

Note: Reported sensitivities for metastatic detection vary across studies and are influenced by reference standards, which are rarely histopathologic for bone and nodal disease.

**Table 6:** Evidence level and clinical readiness of key imaging modalities in prostate cancer prognosis.

Modality	Evidence Level	Clinical Readiness	Guideline Recommendation
mpMRI	Largely validated; prospective RCT data [18,19]	Standard of care	Local staging, biopsy targeting, and active surveillance monitoring [5,66]
WB-MRI	Validated in selected studies; prospective comparative data [117]	Guideline-supported in selected centres	M-staging in high-risk PCa when PSMA PET/CT is unavailable [66]
PSMA PET/CT	Largely validated; prospective multicenter data [12]	Guideline option	Preferred for BCR localization and staging of high- and very-high-risk primary PCa [2,66]
Bone Scintigraphy	Guideline-endorsed; long-standing evidence base [23,66]	Standard of care; widely available	Bone staging where PSMA PET/CT is unavailable; superseded by PSMA PET where accessible [2,66]
CT + Contrast	Guideline-endorsed [2]	Standard of care	Visceral and nodal staging when PSMA PET/MRI unavailable; body composition prognostication in mCRPC [2,66]

Evidence Level: Guideline-endorsed = supported by large prospective or registry studies and incorporated in major clinical guidelines; Largely validated = supported by prospective or large retrospective multicenter studies. Clinical Readiness: Standard of care = guideline-mandated and widely available; Guideline-supported in selected centers = recommended but institutional availability varies.

**Table 7:** Comparison of prognostic imaging modalities and radiotracers in prostate cancer. Abbreviations: S-RT, salvage RT; S-PLND, salvage pelvic lymph node dissection.

Modality/Tracer	Key Prognostic Metrics	Strengths	Limitations
**^18^F- or ^68^Ga-PSMA PET/CT**	SUV_max_, PSMA-TV, TL-PSMA, miTNM stage	High sensitivity and specificity for nodal/bone metastases, stratifies oligometastatic disease	False-negatives in low-PSMA tumors, affected by ADT timing and tumor biology [109,180]
**^18^F-PSMA-1007 PET/CT**	PSMA-TV, bone lesion burden, PSA kinetics	High resolution for bone metastases, less renal excretion than ^68^Ga tracers	Low specificity for benign bone lesions, image interpretation complexity [194]
**^11^C-choline PET/CT**	LN burden, BCR localization, survival after S-RT/S-PLND	Effective in recurrent PCa, impacts salvage RT decisions	Short half-life, lower sensitivity than PSMA for early disease [80,172]
**^18^F-FDG PET/CT**	SUV_max_, intraprostatic uptake, metabolic activity	Useful in high-grade or NEPC, detects PSMA-negative disease	Low sensitivity in low-grade PCa; non-specific uptake in inflammatory or infectious tissues (e.g., prostatitis) may mimic malignancy [165,166,195]
**PSMA PET/MRI**	PSMA uptake, PI-RADS score [196]	Improved local staging, superior soft-tissue contrast, combined metabolic and mpMRI prognostic information [196]	Limited availability, longer acquisition time, higher cost compared with PET/CT [149,150,181]
**CT (Body Composition)**	SAT, VAT, SMM	Non-invasive; SAT and sarcopenia independently prognostic in mCRPC [159,160,162]	Not PCa-specific; indirect prognostic information without tumor visualization [159]
**CBCT Radiomics**	Radiomic score, Gleason grade, BCR prediction	Accessible during RT, non-invasive prognosis monitoring	Still exploratory; standardization required [163]
**^177^Lu-PSMA SPECT/CT**	TTV, NLs, PFS, OS	Effective in mCRPC treatment monitoring	Limited role outside theranostic treatment monitoring [193]; low spatial resolution

**Table 8:** Evidence level and clinical readiness of PET tracers and hybrid modalities in prostate cancer prognosis.

Modality/Tracer	Evidence Level	Clinical Readiness	Primary Clinical Use
^18^F- or ^68^Ga-PSMA PET/CT	Largely validated; prospective multicenter RCT [12]	Guideline option	BCR localization; staging of high- and very-high-risk primary PCa; theranostic patient selection [2,66]
^18^F-PSMA-1007 PET/CT	Emerging; observational and comparative data with known interpretation challenges from indeterminate bone lesions [194,197]	Emerging—guideline-supported in select settings	Alternative to ^68^Ga-PSMA where unavailable; bone-dominant metastatic disease [66]
^11^C-Choline PET/CT	Largely validated; long clinical track record [80,172]	Emerging	BCR evaluation where PSMA PET unavailable; salvage radiotherapy planning [80,172]
^18^F-FDG PET/CT	Largely validated; prospective cohort data [21,166]	Emerging—guideline-supported in select settings	mCRPC with suspected dedifferentiation or neuroendocrine features; discordance assessment for PSMA-targeted therapy [66]
PSMA PET/MRI	Emerging; single-centre and comparative studies [181,196]	Research/selected centres	High-risk primary staging and local recurrence assessment where both mpMRI and PSMA PET are indicated
CT Body Composition	Emerging; retrospective cohort data [159]	Research/selected centres	Prognostic stratification in mCRPC; identifying sarcopenia and metabolic risk
CBCT Radiomics	Investigational; single-centre pilot data [163]	Research only	BCR prediction and ISUP grading during external beam radiotherapy
^177^Lu-PSMA SPECT/CT	Emerging; within theranostic treatment context [21,22,193]	Research/selected centres	Treatment response monitoring in mCRPC during PSMA-targeted radioligand therapy

Evidence Level: Largely validated = supported by prospective or large retrospective multicenter studies; Emerging = predominantly single-center or pilot data; Investigational = no prospective validation. Clinical Readiness: Guideline option = recommended in major guidelines for specific indications; Research/selected centers = not yet in routine clinical guidelines.

**Table 9:** Summary of ultrasound modalities and their prognostic applications in prostate cancer.

Modality	Prognostic Utility	Key Metrics/ Findings	Strengths	Limitations	References
TRUS	Initial grading, biopsy targeting	AUC = 0.85 for patients with <4-year survival	Widely available	Low specificity without MRI fusion	[13,199]
CEUS	Risk stratification, recurrence prediction	AUC = 0.910 (training), 0.879 (validation); TIC metrics such as PI, TTP	Visualizes tumor vascularity; improves biopsy accuracy	Operator-dependent; requires contrast agent; not broadly used in the clinics	[201,202]
SWE	Tumor grade correlation	r=0.74, p<0.001 (tissue stiffness); SHG: rs=0.93	Quantifies stiffness non-invasively	Requires further validation	[206]
MicroUS	Index lesion detection, risk classification	Comparable to mpMRI; superior for tumor extent in some studies	High spatial resolution; cost-effective alternative to MRI; commercially available systems; compatible with MRI fusion–guided targeting.	Still emerging in clinical practice	[203,205]

Abbreviations: TRUS, transrectal ultrasound; AUC, area under the receiver operating characteristic curve; CEUS, contrast-enhanced ultrasound; TIC, time–intensity curve; PI, peak intensity; TTP, time-to-peak; SWE, shear wave elastography; SHG, second-harmonic generation; MicroUS, micro-ultrasound; mpMRI, multiparametric MRI.

## Data Availability

No new data were created in this study. Data sharing is not applicable to this article.

## Abbreviations

The following abbreviations are used in this manuscript:

MRI	Magnetic Resonance Imaging
CT	Computed Tomography
PET	Positron Emission Tomography
TRUS	Transrectal Ultrasound
PET/MRI	Positron Emission Tomography/Magnetic Resonance Imaging
PET/CT	Positron Emission Tomography/Computed Tomography
PSA	Prostate-Specific Antigen
mpMRI	Multiparametric MRI
PSMA	Prostate-Specific Membrane Antigen
PCa	Prostate Cancer
NICE	National Institute for Health and Care Excellence
EAU	European Association of Urology
AUA	American Urological Association
NCCN	National Comprehensive Cancer Network
MSKCC	Memorial Sloan Kettering Cancer Center
CAPRA	Cancer of the Prostate Risk Assessment
CPG	Cambridge Prognostic Groups
csPCa	Clinically Significant Prostate Cancer
CRPC	Castration-Resistant Prostate Cancer
mCRPC	Metastatic Castration-Resistant Prostate Cancer
^68^Ga-PSMA	Gallium-68–labeled PSMA
BCR	Biochemical Recurrence
RT	Radiotherapy
ADT	Androgen Deprivation Therapy
MFS	Metastasis-Free Survival
PCSM	Prostate Cancer-Specific Mortality
OS	Overall Survival
RP	Radical Prostatectomy
PSM	Positive Surgical Margins
EBRT	External Beam Radiotherapy
ALP	Alkaline Phosphatase
LDH	Lactate Dehydrogenase
Fib	Fibrinogen
OR	Odds Ratio
NLR	Neutrophil-to-Lymphocyte Ratio
CRP	C-reactive Protein
NPCC	Norwegian Prostate Cancer Consortium
DREAM	Dialogue for Reverse Engineering Assessments and Methods
ePCR	Ensemble Prostate Cancer Risk model
HR	Hazard Ratio
CI	95% Confidence Interval
CRISP3/SPINK1	Cysteine-Rich Secretory Protein 3/Serine Peptidase Inhibitor Kazal-Type 1
CSPG	CRISP3/SPINK1 Prognostic Grade
AUC	Area Under the Receiver Operating Characteristic Curve
AS	Active Surveillance
RSI-MRI	Restriction Spectrum Imaging–MRI
PHI	Prostate Health Index
PSAD	PSA Density
PI-RADS	Prostate Imaging Reporting and Data System
PI-RR	Prostate Magnetic Resonance Imaging for Local Recurrence Reporting
LNI	Lymph Node Involvement
ISUP	International Society of Urological Pathology
GG	Grade Group
TCA	Tricarboxylic Acid
VEGF	Vascular Endothelial Growth Factor
IL-6	Interleukin-6
MiPS2	Michigan Prostate Score 2
PCA3	Prostate Cancer Antigen 3
SPDEF	SAM Pointed Domain-Containing ETS Transcription Factor
TMPRSS2-ERG	Transmembrane Protease, Serine 2–ETS-Related Gene Fusion
RNA	Ribonucleic Acid
IDC-P	Intraductal Carcinoma of the Prostate
CSS	Cancer-Specific Survival
DFS	Disease-Free Survival
SEER	Surveillance, Epidemiology, and End Results
bDFS	Biochemical Disease-Free Survival
HRLG	High-Risk Low Grade
HRHG	High-Risk High Grade
au-lncRNAs	Autophagy-related Long Non-Coding RNAs
ML	Machine Learning
SNP	Single-Nucleotide Polymorphism
TCGA-PRAD	The Cancer Genome Atlas – Prostate Adenocarcinoma
PCDI	Programmed Cell Death Index
BRCA1/2	Breast Cancer gene 1 and 2
ATM	Ataxia Telangiectasia Mutated
CHEK2	Checkpoint Kinase 2
AR	Androgen Receptor
PTEN/PI3K-AKT	Phosphatase and Tensin Homolog/Phosphoinositide
	3-Kinase–Protein Kinase B
PARP	Poly(ADP-ribose) Polymerase
ctDNA	Circulating Tumor DNA
WB-MRI	Whole-Body Magnetic Resonance Imaging
DCE	Dynamic Contrast-Enhanced
DWI	Diffusion-Weighted Imaging
AI	Artificial Intelligence
3D T2WI	3D T2-Weighted Imaging
ADC	Apparent Diffusion Coefficient
EPE	Extraprostatic Extension
SVI	Seminal Vesicle Invasion
99mTc	Technetium-99m
BS	Bone Scintigraphy
rFF%	Relative Fat Fraction
MRSI	Magnetic Resonance Spectroscopic Imaging
FLARE	A transient increase in osteoblastic activity on bone scans
	following effective treatment
TXR	Targeted X-rays
PFS	Progression-Free Survival
RFS	Recurrence-Free Survival
bp-MRI	Biparametric MRI
SNR	Signal-to-Noise Ratios
18F-PSMA-1007	18F-labeled Prostate-Specific Membrane Antigen ligand 1007
PSMA-TV	PSMA-Tumor Volume
TL-PSMA	Total Lesion PSMA
PSMA-TTL	Whole-body Total Lesion PSMA Burden
Dmax	Maximum Dissemination Distance
11C-choline	11C-labeled Choline
NEPC	Neuroendocrine Prostate Cancer
FDG	Fluorodeoxyglucose
SUVmax	Maximum Standardized Uptake Value
SAT	Subcutaneous Adipose Tissue
VAT	Visceral Adipose Tissue
SMM	Skeletal Muscle Mass
CBCT	Cone Beam CT
ASTRO	American Society for Therapeutic Radiology and Oncology
[18F]DCFPyL	18F-labeled PSMA ligand
TLA	Total Lesion Activity
MTV	Molecular Tumor Volume
TLR	Tumor-to-Liver Ratio
IBSI	Image Biomarker Standardisation Initiative
PROMISE	Prostate Cancer Molecular Imaging Standardized Evaluation
TNM	Tumor-Node-Metastasis (staging)
miTNM	Molecular Imaging TNM (staging)
PSMA-RADS	PSMA Reporting and Data System
SPECT/CT	Single-Photon Emission Computed Tomography/Computed Tomography
177Lu-PSMA-617	Lutetium-177–labeled Prostate-Specific Membrane Antigen–617
TTV	Total Tumor Volume
NLs	New Lesions
S-RT	Salvage Radiotherapy
S-PLND	Salvage Pelvic Lymph Node Dissection
HIFU	High-Intensity Focused Ultrasound
CEUS	Contrast-Enhanced Ultrasound
microUS	Micro-ultrasound
TPV	Total Prostate Volume
BCR-FS	Biochemical Recurrence-Free Survival
qCEUS	Quantitative CEUS
WoAUC	Wash-Out Area Under the Curve
TIC	Time-Intensity Curve
PI	Peak Intensity
TTP	Time-to-Peak
SWE	Shear Wave Elastography
SHG	Second-Harmonic Generation
SEU	Sonazoid-Enhanced Ultrasound
HNRNPC	Heterogeneous Nuclear Ribonucleoprotein C
CHAARTED	ChemoHormonal Therapy versus Androgen Ablation Randomized
	Trial for Extensive Disease
ECE	Extracapsular Extension
GPS	Genomic Prostate Score
HRR	Homologous Recombination Repair
MDT	Multidisciplinary Team
mHSPC	Metastatic Hormone-Sensitive Prostate Cancer
PRECISE	Prostate Cancer Radiological Estimation of Change in Sequential Evaluation
